# Second sacral sacralalar‐iliac (S2AI) screw placement in adult degenerative scoliosis (ADS) patients: an imaging study

**DOI:** 10.1186/s12893-021-01139-w

**Published:** 2021-04-06

**Authors:** Bing Wu, Kai Song, Junyao Cheng, Pengfei Chi, Zhaohan Wang, Zheng Wang

**Affiliations:** Department of Orthopedics, the Fourth Medical Centre, Chinese PLA General Hospital, 51 Fucheng Road, Haidian District, 100048 Beijing, China

**Keywords:** S2AI screw, Adult degenerative scoliosis, Spinal fusion, Pelvis, Screw trajectory

## Abstract

**Background:**

The imaging characteristics of sacral sacralalar-iliac (S2AI) screw trajectory in adult degenerative scoliosis (ADS) patients will be determined.

**Methods:**

S2AI screw trajectories were mapped on three-dimensional computed tomography (3DCT) reconstructions of 40 ADS patients. The starting point, placement plane, screw template, and a circle centered at the lowest point of the ilium inner cortex were set on these images. A tangent line from the starting point to the outer diameter of the circle was selected as the axis of the screw trajectory. The related parameters in different populations were analyzed and compared.

**Results:**

The trajectory length of S2AI screws in ADS patients was 12.00 ± 0.99 cm, the lateral angle was 41.24 ± 3.92°, the caudal angle was 27.73 ± 6.45°, the distance from the axis of the screw trajectory to the iliosciatic notch was 1.05 ± 0.81 cm, the distance from the axis of the screw trajectory to the upper edge of the acetabulum was 1.85 ± 0.33 cm, and the iliac width was 2.12 ± 1.65 cm. Compared with females, the lateral angle of male ADS patients was decreased, but the trajectory length was increased (*P* < 0.05). Compared to patients without ADS in previous studies, the lateral angle of male patients was larger, the lateral angle of female patients was increased, and the caudal angle was decreased (*P* < 0.05).

**Conclusions:**

There is an ideal trajectory of S2AI screws in ADS patients. A different direction should be noticed in the placement of S2AI screws, especially in female patients.

## Background

With aging of the population, the overall incidence of adult degenerative scoliosis (ADS) is 8.3–68 % [1, 2]. ADS is a three-dimensional deformity of the spine that is often accompanied by morphologic changes (pelvic retroversion and coronal tilt) [3, 4]. Long segmental fixation is often required when coronal and sagittal imbalances are caused by severe deformities. Surgical treatment is thought to be the only way to relieve symptoms in ADS patients who fail to respond to conservative treatment.

Surgeons face severe challenges for long-segment fixed fusion to S1 in ADS patients. Specifically, the S1 vertebral body has a structural disadvantage that is characterized by short and thick pedicles and a stress concentration is noted in the lumbosacral region after long segment fixation. In addition, ADS patients often have co-existing osteoporosis, which leads to a decrease in bone-screw interface control, resulting in poor lumbosacral fusion, false joint formation, and even orthopedic failure [5–7]. Moreover, the incidence of complications is higher when long-segment fixation is fused to S1. Kim [8] reported that the rate of screw loosening in adult spinal deformities is 24.4 % after distal fusion to S1 alone. Emami [9] showed that the non-fusion rate of lumbosacral is approximately 20 % when long-segment fusion reaches the sacrum alone. Kim [10] found that the fusion rate of lumbosacral pseudoarthroplasty with long-segment fixation is 17 %. Therefore, it is necessary to extend the fixation to the pelvis to increase the mechanical strength of the lumbosacral region.

Compared with the traditional spinal and pelvic fixation technology, the sacral sacralalar-iliac (S2AI) screw fixation technology has the advantages of less tissue dissection, less trauma, small incision, high fixation strength, and good compliance with a lumbosacral pedicle screw [11–15]. In recent years, the trajectory of the S2AI screw and evaluation of its clinical effect have become major research interests. Previous studies on S2AI screw trajectories at home and abroad are all derived from 3DCT measurements or cadaver studies in healthy people and patients with lumbar degenerative disc disease [11, 12, 16–18].

The pelvic morphology of the ADS population might be different from the healthy population or patients with lumbar spine disease. Therefore, the data obtained from the latter two populations would be appropriate for the target population that needs S2AI screw insertion. In addition, a study of the S2AI screw trajectory based on the sample of ADS patients has not been reported. In this study the trajectory of S2AI screws was standardized by 3DCT reconstruction images of the lumbosacral pelvic region in ADS patients, the related parameters were measured and analyzed to provide the imaging anatomic basis of S2AI screw fixation, and the characteristics of S2AI screw trajectory in ADS patients were summarized.

## Methods

### Inclusion and exclusion criteria

The study had been approved by the Ethics Committee of our hospital and was conducted under the requirements for good clinical practice. A total of 40 patients with degenerative scoliosis who were admitted to our department between September 2015 and December 2016 were selected for imaging measurements. The inclusion criteria included the following: (1) patients ≥ 50 years of age diagnosed with lumbar degenerative scoliosis and a Cobb angle of at least 10°. (2) The design of the S2AI screw track trajectory was standardized through three-dimensional reconstruction of CT images of the lumbosacral pelvis region, and the relevant parameters were measured and analyzed. The exclusion criteria included the following: 1. Recurrent scoliosis or deformity before skeletal maturity, such as idiopathic, neuromuscular, and congenital scoliosis. 2. Scoliosis caused by spinal trauma, spinal surgery, tumors, or infections. (3) Scoliosis resulting from unequal lengths of the lower limbs or non-primary diseases of the hip, knee, or ankle. (4) Less than 50 years of age. Our study population consisted of 40 patients (15 males and 25 females) with a mean age (and standard deviation) of 65 ± 6.73 years (range, 51–81 years).

Due to CT examination radiation exposure, a control group in the general population without ADS was not established in this study. Therefore, the relevant literature of S2AI screws in domestic and foreign databases was retrieved as the control group. The literature inclusion criteria were as follows: complete main parameters of screw trajectory, such as screw trajectory length, caudal angle, and lateral angle, were measured and all data are expressed as − x ± s. A total of 4 studies were retrieved [19–22].

### Standardized design of S2AI screw trajectory

Three-dimensional scanning of the thoracolumbar, lumbar, and sacral segments of the spine was performed with a sliding mode 40 slice spiral CT scanning (Somatom Sensation 40; Siemens, Erlangen, Germany) in our hospital, and the scanning range also included the proximal femur of the bilateral hip joints. After being transmitted to the post-processing workstation of the Syngo software system, image reconstruction and reorganization were carried out with a reconstruction gap of 1.5 mm.

**Fig. 1:**
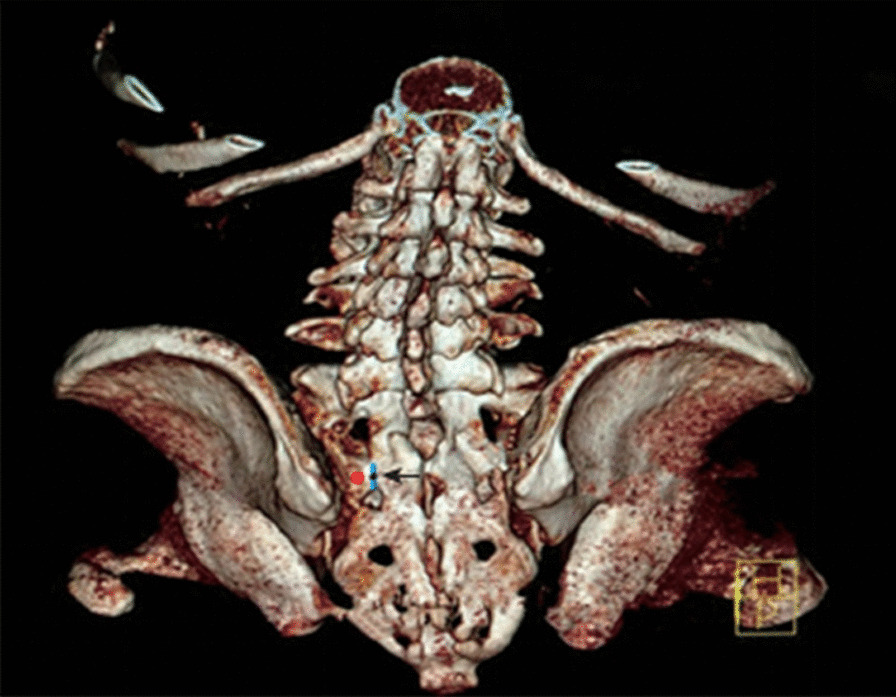
Three-dimensional CT reconstruction of the lumbosacral pelvis: red dot represents the insertion point/ starting point

**Fig. 2:**
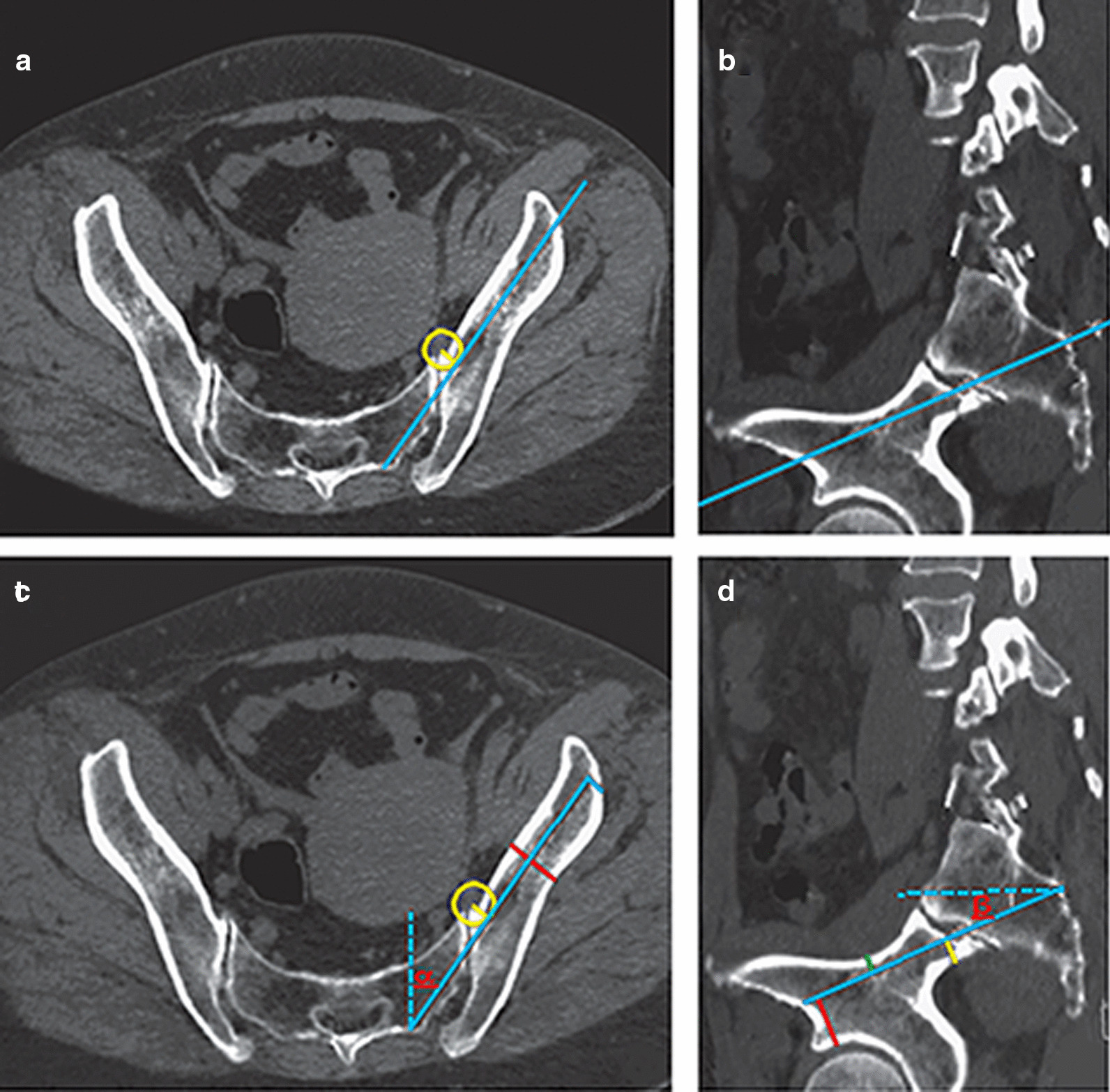
Standardized design of S2AI screw trajectory and measurement of relevant parameters. **a** Transverse image of screw placement plane. The blue line represents the axis of the nail track, and the yellow line represents the distance between the anterior cortex of the ilium and the axis of the screw trajectory by 5 mm. **b** Sagittal image of screw placement plane. The blue line indicates the point of insertion to the inferior margin of anterior inferior iliac spine. **c** Transverse image of screw placement plane. Alpha is the lateral angle of the screw trajectory, the blue line represents the length of the screw trajectory, and the red line represents the width of the ilium. **d** Sagittal image of screw placement plane. Beta is the caudal angle of the screw trajectory, the yellow line represents the distance from the screw trajectory to the ischial notch, the green line represents the distance from the screw trajectory to the upper iliac cortex, and the red line represents the distance from the screw trajectory to the acetabulum

The base of the lateral sacral crest on the midline between the lower edge of the S1 dorsal foramina and the upper edge of the S2 dorsal foramina was designated as the starting point, and was also the entry point of the nail (Fig. 1). The placement plane of the S2AI screw trajectory was determined from the starting point to the lower margin of the anterior inferior iliac spine. A 10-mm diameter screw was the design template and a distance of 0.5 cm from the anterior cortical bone in the iliac bone was designated as the axis of the safe track of the nail path, which was also the best nail path (Fig. 2a, b).

### Measurement of parameters

1. The transverse plane measurement (Fig. 2c) was as follows: (1)The lateral angle was the angle between the projection of the screw trajectory on the cross-section and the median line. (2) The trajectory length was the distance between the starting point and the lateral cortex along the trajectory. (3) The iliac width was the narrowest distance between the inner and outer cortex of the ilium along the screw trajectory.
2. The sagittal plane measurement (Fig. 2d) was as follows: (1) The caudal angle was the angle between the projection of the screw trajectory on the sagittal plane and the horizontal line. (2) The distance from the axis of the screw trajectory to the iliosciatic notch was the shortest vertical distance from the highest point of the ischial large notch to the screw trajectory, which represents the space distance of the screw trajectory below the sagittal plane. (3) The distance from the axis of the screw trajectory to the upper edge of the acetabulum was the shortest vertical distance between the lowest point of the bone above the screw trajectory and represented the space distance of the screw trajectory above the sagittal plane. (4) The distance between the screw trajectory and the acetabulum was the shortest vertical distance between the highest point of the acetabulum and the screw trajectory. The lumbar lordosis, pelvic incidence, pelvic tilt, and sacral slope also were measured.

### Statistical analysis

Statistical analysis was carried out using SPSS 22.0 (SPSS, Inc., Chicago, IL, USA). The measurement parameters are expressed as − x ± s. The differences in parameters between the males and females in our study were analyzed using an independent samples *t*-test. The main parameters of the S2AI screw trajectory were compared with the results reported in the literature using the SNK - *Q* test. A statistically significant difference was considered at a *p* < 0.05.

## Results

### Trajectory parameters of the S2AI screw in ADS patients

The trajectory length of the S2AI screw in all ADS patients was 12.00 ± 0.99 cm, the lateral angle was 41.24° ± 3.92°, the caudal angle was 27.73° ± 6.45°, the distance from the axis of the screw trajectory to the iliosciatic notch was 1.05 ± 0.81 cm, the distance from the axis of the screw trajectory to the upper edge of the acetabulum was 1.85 ± 0.33 cm, and the iliac width was 2.12 ± 1.65 cm.

### Comparison of main parameters in ADS patients of different genders

Because there was no significant difference between the left and right S2AI screw trajectory parameters in patients of the same gender, the main parameter data of the screw trajectory in two sides were combined and analyzed according to different genders. The male lateral angle (39.47° ± 1.76°) decreased by nearly 3° compared with the female lateral angle (42.30° ± 4.48°; *P* < 0.05). The length of the male screw trajectory was 12.40 ± 0.83 cm, while length of the female screw trajectory was 11.75 ± 1.01 cm, which increased by 0.55 cm on average in males compared to females (*P* < 0.05). The differences in caudal angle, lumbar lordosis, pelvic incidence, pelvic tilt and sacral slope between the genders were not statistically significant (Table 1).

**Table 1:** Parameters of S2AI screw in patients with ADS(x ± s)

	Total(40)	Male(15)	Female(25)	*t* value	*P* value
Lateral angle(°)	41.24 ± 3.92	39.47 ± 1.76	42.30 ± 4.48	2.33	0.025
Caudal angle(°)	27.73 ± 6.45	28.00 ± 6.39	27.56 ± 6.61	− 0.21	0.838
Length of trajectory(cm)	12.00 ± 0.99	12.40 ± 0.83	11.75 ± 1.01	− 2.07	0.045

### Comparison of the main parameters of S2AI screw trajectories in ADS patients based on the extant literature

The parameters of S2AI screw trajectories reported in the extant literature were collected and compared (Tables 2, 3, 4, 5, 6, and 7 ). The length of S2AI screw trajectories in ADS patients was longer, but there was no significant difference compared with previous publications (Tables 2 and 3).

**Table 2:** The length of the trajectory in male patients with ADS compared with that reported in the previous literature

	Sample size	Total(cm)	Left(cm)	Right(cm)	*t* value^a^	*P* value^a^
Current study	15	12.40 ± 0.83	12.38 ± 0.86	12.42 ± 0.84	–	–
Yamada etc. [19]	40	–	12.15 ± 10.30	12.18 ± 10.10	left: − 0.09; right: – 0.09	left: 0.932;right: 0.928
Zhu etc. [20]	30	–	12.13 ± 8.33	12.06 ± 7.54	left: − 0.12; right: – 0.18	left: 0.909;right: 0.856
Yuan etc. [21]	25	12.09 ± 7.89	–	–	− 0.15	0.881
Wang etc. [22]	30	12.00 ± 6.52	–	–	− 0.24	0.815

^a^The statistical values are the data in each literature compared with the corresponding data in this study, the same below

**Table 3:** The length of the trajectory in female patients with ADS compared with that reported in the previous literature

	Sample size	Total(cm)	Left(cm)	Right(cm)	*t* value^a^	*P* value^a^
Current study	25	11.75 ± 1.01	11.75 ± 1.02	11.76 ± 1.08	−	−
Yamada etc. [19]	40	−	11.38 ± 9.60	11.27 ± 9.10	left: − 0.19;right: − 0.27	left: 0.849;right: 0.790
Zhu etc. [20]	30	−	11.48 ± 9.44	11.57 ± 8.24	left: − 0.14;right: − 0.11	left: 0.888;right: 0.909
Yuan etc. [21]	25	11.52 ± 8.80	−	−	– 0.13	0.897
Wang etc. [22]	30	10.95 ± 4.63	−	−	– 0.85	0.399

^a^The statistical values are the data in each literature compared with the corresponding data in this study, the same below

**Table 4:** The caudal angle in male patients with ADS compared with that reported in the previous literature

	Sample size	Total(°)	Left(°)	Right(°)	*t* value ^a^	*P* value ^a^
Current study	15	28.00 ± 6.39	27.73 ± 6.68	28.27 ± 6.10	−	−
Yamada etc. [19]	40	−	27.65 ± 6.80	28.00 ± 7.20	left: − 0.11;right: 0.13	left: 0.911;right: 0.898
Zhu etc. [20]	30	−	29.15 ± 8.60	29.96 ± 8.28	left: − 0.56;right: − 0.70	left: 0.579;right: 0.489
Yuan etc. [21]	25	29.56 ± 8.38	−	−	0.62	0.539
Wang etc. [22]	30	31.02 ± 7.43	−	−	1.39	0.170

^a^The statistical values are the data in each literature compared with the corresponding data in this study, the same below

**Table 5:** The lateral angle in male patients with ADS compared with that reported in the previous literature

	Sample size	Total(°)	Left(°)	Right(°)	*t* value^a^	*P* value^a^
Current study	15	39.47 ± 1.76	39.60 ± 1.99	39.33 ± 2.02	−	−
Yamada etc. [19]	40	−	37.90 ± 7.00	37.70 ± 7.50	left: − 0.092;right: − 0.83	left: 0.361;right: 0.412
Zhu etc. [20]	30	−	36.49 ± 3.14	37.16 ± 3.14	left: − 3.49;right: − 2.43	left: 0.001;right: 0.019
Yuan etc. [21]	25	36.11 ± 3.38	−	−	– 3.56	0.001
Wang etc. [22]	30	40.25 ± 2.84	−	−	0.99	0.326

^a^The statistical values are the data in each literature compared with the corresponding data in this study, the same below

**Table 6:** The caudal angle in female patients with ADS compared with that reported in the previous literature

	Sample size	Total(°)	Left(°)	Right(°)	*t* value^a^	*P* value^a^
Current study	25	27.56 ± 6.61	27.68 ± 6.30	27.44 ± 7.71	−	−
Yamada etc. [19]	40	−	33.40 ± 6.40	33.90 ± 6.60	left: 3.53;right: 3.60	< 0.001
Zhu etc. [20]	30	−	34.50 ± 6.56	35.72 ± 7.53	left: 3.91;right: 4.02	< 0.001
Yuan etc. [21]	25	35.11 ± 7.03	−	−	3.91	< 0.001
Wang etc. [22]	30	34.16 ± 6.02	−	−	4.14	< 0.001

^a^The statistical values are the data in each literature compared with the corresponding data in this study, the same below

**Table 7:** The lateral angle in female patients with ADS compared with that reported in the previous literature

	Sample size	Total(°)	Left(°)	Right(°)	*t* value^a^	*P* value^a^
Current study	25	42.30 ± 4.48	41.92 ± 5.10	42.68 ± 3.98	−	−
Yamada etc. [19]	40	−	32.80 ± 7.60	32.40 ± 7.10	left: − 5.29;right: − 6.61	< 0.001
Zhu etc. [20]	30	−	35.72 ± 3.79	36.27 ± 3.27	left: − 5.17;right: − 6.56	< 0.001
Yuan etc. [21]	25	36.69 ± 3.21	−	−	5.09	< 0.001
Wang etc. [22]	30	39.25 ± 2.64	−	−	3.46	< 0.001

^a^The statistical values are the data in each literature compared with the corresponding data in this study, the same below

In the current study the lateral angle of the male ADS patients was 39.47° ± 1.76°, which was greater than the healthy population previously reported (*P* < 0.05). The caudal angle was 28.00° ± 6.39° and there was no significant difference between the male ADS patients and the healthy population (Tables 4 and 5).

The caudal angle of the female ADS patients was 27.56° ± 6.61°, which was significantly less than healthy people previously reported (*P* < 0.05). The lateral angle was 42.30° ± 4.48°, which was significantly greater than healthy people (*P* < 0.05; Tables 6 and 7).

## Discussion

The screw entry points of S2AI based on a review of the literature are not fixed positions, but relatively similar to each other [16, 19–28]. The medial base of the lateral sacral ridge on the horizontal line between the lower and upper edges of the posterior sacral foramen of S2 is considered as the entry point, which is similar to the entry point selected by Yamada et al. [19] and William and Jeffery [29]. Indeed, the medial base of the lateral sacral ridge is the key to determining the trajectory plane of the S2AI screw, and at the same time, the length and width of the internal part of the ilium must be considered. Thus, the line from the most convex point of the posterior superior iliac spine to the anterior inferior iliac spine is the longest diameter line in the ilium [30], and the medullary cavity is wider toward the caudal end of the ilium [31]. In the current study the location of the entry point was close to the posterior superior iliac spine, and the length of the screw trajectory and the safety of screw placement were considered. Therefore, the direction of the screw should be toward the inferior margin of the anterior inferior iliac spine, which simplifies the steps to identify the screw placement plane, which was confirmed by constantly rotating the image in previous studies [20–22]. Of note, the measurement method had little effect on the differences in each study. In the current study, a 10-mm diameter screw was used as the design template. The more forward the S2AI screw trajectory in the cross-section, the longer the screw trajectory. Based on this feature, the 5-mm distance from the anterior cortex bone in the ilium, which is applicable to the diameter screws in clinical practice, was taken as the safety trajectory in the current study. Our results showed that the selected iliac plane was relatively wide and thick. The distance between the sagittal screw trajectory and the ischial notch, the distance between the screw trajectory and the iliac cortex, and the distance between the screw trajectory and the acetabulum were all > 5 mm, thus the location of the S2AI screw was in the bone structure of both the cross-section and sagittal plane, which is safe and reliable.

The results of the current study showed that there was no significant difference in the length of the screw trajectory between the male and female patients with ADS compared with previous studies, which means the data of S2AI screws in our study and previous studies are suitable for ADS patients. In addition to the length of the screw trajectory, the direction of S2AI screw placement in females with ADS was quite different from the previous studies. The results of this study showed that the caudal angle of female ADS patients was significantly less (6–8°) than that reported in the literature, while the lateral angle was significantly greater (3–6°) than that reported in the literature (P < 0.05). The larger lateral angle was mainly due to the difference in pelvis morphology between ADS patients and the general population. The difference in pelvis morphology is not only the cause of ADS, but also the result of ADS. The larger lateral angle of ADS in women suggests that the extroversion should increase as much as possible when inserting S2AI screws. According to previous data, the angle of screw placement is often small, which results in the penetration of the medial cortex of the pelvis and the injury of pelvic organs and blood vessels. In addition, the change in screw trajectory will be increasingly difficult. Because the majority of ADS patients are women, attention should focus on the increased lateral angle. In contrast, the caudal angle of ADS in female patients was smaller than the healthy population, and was related to the overall sagittal imbalance and the lumbar lordosis angle reduction in women with ADS. The loss of lumbar lordosis in ADS patients indicates that the pelvis is tilted backward, which is equal to the reduction of caudal angle in the horizontal position. In spite of the differences in lumbar lordosis of the prone and standing positions, there is still a close relationship between the two positions [32, 33].Therefore, the caudal angle reported in the extant literature is actually based on the general population with normal spine pelvis sagittal parameters. This finding suggests that when S2AI screws are inserted in clinical practice, the caudal angle should be relatively reduced to avoid injuring other tissues by cutting out the large ischial incision.

In the current study the lateral angle of male ADS patients was larger than the healthy population reported in some studies (P < 0.05), but there was no significant difference in the caudal angle. Based on our analysis we believe that the severe sagittal imbalance of the spine pelvis in male patients was less than female patients, and the same was true in male patients with ADS included in this study; however, it was difficult to reach a conclusion similar to that in female ADS patients due to the small sample size. In this paper there were some shortcomings. Because the subjects in this study were ADS patients with detailed CT information of the pelvis, it was impossible to obtain a larger sample size. In addition, although the relevant research suggests that the prone and supine positions are closely related in pelvis rotation, there is still a lack of CT data in the supine position to simulate the state of the prone position intraoperatively, as mainly reflected by the caudal angle.

## Conclusions

In conclusion, there is an ideal trajectory of S2AI screws in ADS patients. There was no statistical significance in the difference of the S2AI screw length between ADS patients and the non-ADS population. A different direction should be noted in the placement of S2AI screws, especially in female ADS patients in whom the lateral angle should increase and the caudal angle should decrease.

## Data Availability

The datasets used and/or analyzed during the current study are available from the corresponding author upon reasonable request.

## Abbreviations

S2AI: second sacral sacralalar-iliac
ADS: adult degenerative scoliosis
3DCT: three-dimensional computed tomography
